# Cardiovascular magnetic resonance evidence of myocardial fibrosis and its clinical significance in adolescent and adult patients with Ebstein’s anomaly

**DOI:** 10.1186/s12968-018-0488-1

**Published:** 2018-09-27

**Authors:** Dan Yang, Xiao Li, Jia-Yu Sun, Wei Cheng, Andreas Greiser, Tian-Jing Zhang, Hong Liu, Ke Wan, Yong Luo, Qi An, Yiu-Cho Chung, Yuchi Han, Yu-Cheng Chen

**Affiliations:** 1 Department of Cardiology, West China Hospital, Sichuan University, Chengdu, 610041 Sichuan Province China; 2 Department of Cardiovascular Surgery/Pediatric Heart Center, West China Hospital, Sichuan University, Chengdu, 610041 Sichuan Province China; 3 Department of Radiology, West China Hospital, Sichuan University, Chengdu, 610041 Sichuan Province China; 4 Siemens Healthcare GmbH, Erlangen, Germany; 5 Northeast Asia MR Collaboration, Siemens Healthcare, Beijing, China; 6 Paul C. Lauterbur Research Centre for Biomedical Imaging, Shenzhen Key Laboratory for MRI, Shenzhen Institutes of Advanced Technology, Chinese Academy of Sciences, Shenzhen, Guangdong China; 7 Department of Medicine (Cardiovascular Division), University of Pennsylvania, Philadelphia, PA USA

**Keywords:** Ebstein’s anomaly, Cardiovascular magnetic resonance imaging, Extracellular volume, Late gadolinium enhancement, Left ventricular function, Myocardial fibrosis

## Abstract

**Background:**

Myocardial fibrosis is a common pathophysiological process that is related to ventricular remodeling in congenital heart disease. However, the presence, characteristics, and clinical significance of myocardial fibrosis in Ebstein’s anomaly have not been fully investigated. This study aimed to evaluate myocardial fibrosis using cardiovascular magnetic resonance (CMR) late gadolinium enhancement (LGE) and T1 mapping techniques, and to explore the significance of myocardial fibrosis in adolescent and adult patients with Ebstein’s anomaly.

**Methods:**

Forty-four consecutive patients with unrepaired Ebstein’s anomaly (34.0 ± 16.2 years; 18 males), and an equal number of age- and gender-matched controls, were included. A comprehensive CMR protocol consisted of cine, LGE, and T1 mapping by modified Look-Locker inversion recovery (MOLLI) sequences were performed. Ventricular functional parameters, native T1, extracellular volume (ECV), and LGE were analyzed. Associations between myocardial fibrosis and disease severity, ventricular function, and NYHA classification were analyzed.

**Results:**

LGE was found in 10 (22.7%) patients. Typical LGE in Ebstein’s anomaly was located in the endocardium of the septum within the right ventricle (RV). The LV ECV of Ebstein’s anomaly were significantly higher than those of the controls (30.0 ± 3.8% vs. 25.3 ± 2.3%, *P <* 0.001). An increased ECV was found to be independent of the existence of LGE. Positive LGE or higher ECV (≥30%) was associated with larger fRV volume, aRV volume, increased disease severity, and worse NYHA functional class. In addition, ECV was significantly correlated with the LV ejection fraction (*P* <  0.001).

**Conclusions:**

Both focal and diffuse myocardial fibrosis were observed in adolescent and adult patients with Ebstein’s anomaly. Increased diffuse fibrosis is associated with worse LV function, increased Ebstein’s severity, and worse clinical status.

## Background

Ebstein’s anomaly is a rare congenital heart disease. It is considered a right ventricular (RV) myopathy with failure of delamination of the tricuspid leaflets. Ebstein's anomaly is associated with considerable tricuspid regurgitation and the formation of an atrialized right ventricle (aRV) [1–4]. Compromised RV function, in varying degrees, is frequently found in Ebstein's anomaly, and it is believed to be the major cause of heart failure [2, 3, 5, 6].

Compared with the tricuspid valve and RV abnormalities, the left ventricle (LV) of Ebstein’s anomaly is less studied. LV dysfunction was found in 7–50% patients with Ebstein’s anomaly and was associated with worse prognosis after surgery [7–10]. The mechanism of LV dysfunction in Ebstein’s anomaly is yet to be explored. Myocardial fibrosis was found in the left heart of decedents with Ebstein’s anomaly [11, 12]. Limited by the available techniques, to date, it has been difficult to explore the relationship between myocardial fibrosis and ventricular dysfunction in vivo.

Cardiovascular magnetic resonance (CMR) is a powerful non-invasive imaging modality to evaluate myocardial fibrosis. Late gadolinium enhancement (LGE) is an established CMR technique that provides accurate quantitation of myocardial replacement fibrosis. Recent advances in CMR native T1 mapping have provided us with a groundbreaking tool for quantification of diffuse myocardial fibrosis. The extracellular volume fraction (ECV) calculated from pre and post contrast T1 mapping images showed a strong correlation with collagen volume fraction histologically obtained from endomyocardial biopsies [13, 14]. Myocardial LGE is strongly related to myocardial viability, ventricular function, and adverse prognosis in patients with ischemic heart disease, dilated cardiomyopathy, hypertrophic cardiomyopathy, and cardiac amyloidosis [15–18]. In congenital heart disease, the existence of LGE located in the RV outflow tract is associated with the occurrence of ventricular tachycardia in patients with repaired Tetralogy of Fallot [19]. T1 mapping also revealed diffuse fibrosis in repaired Tetralogy of Fallot and pulmonary hypertension [20, 21]. With the availability of these techniques, we are now able to study myocardial fibrosis and its significance in patients with Ebstein’s anomaly. Therefore, in the present study, we employed LGE and T1 mapping techniques to quantify focal and diffuse myocardial fibrosis in patients with Ebstein’s anomaly, and to investigate their correlations with the severity of Ebstein’s anomaly, ventricular function, and clinical status.

## Methods

### Study population

Consecutive patients with unrepaired Ebstein’s anomaly referred to our institution for clinical evaluation gave their consent and were enrolled into a registry since June 2013. All patients received a comprehensive evaluation including a detailed medical history, physical examination, oxygen saturation test, routine blood examination (including serum creatine), electrocardiography, echocardiography, and CMR imaging. Patients in our registry database from June 2013 to Oct 2016 were reviewed and patients with complete data sets and those older than ten years of age were included in the study. Exclusion criteria were as follows: 1) Contraindications to contrast-enhanced CMR; 2) other coexisting cardiomyopathy, such as hypertrophic, restrictive, or infiltrative cardiomyopathy; 3) a history of known coronary heart disease or myocarditis; 4) other complex congenital cardiac anomalies (e.g., congenitally corrected transposition with Ebstein’s anomaly); 5) CMR image quality was too poor to analyze due to significant arrhythmia or difficulty with breath-holding. Patients were grouped into Carpentier’s classification of types A to D, based on the mobility of the tricuspid leaflet and the RV shape and contractility on echocardiography [22].

An equal number of age- and gender-matched subjects were included as controls. Healthy adults with no documented cardiovascular diseases or other major illnesses, and with normal echocardiograms, were recruited as volunteers. Subjects younger than 18 years old were selected for CMR examination to screen for myocarditis. Subjects were included as controls if all clinical data, routine blood work, electrocardiography, echocardiography, and CMR examination excluded cardiovascular disease. All patients and controls (or their parents if a subject was younger than 18 years old) gave written informed consent for their data to be included in this study.

### CMR data acquisition

CMR was performed on a 3 T scanner (Magnetom Tim Trio, Siemens Healthineers, Erlangen, Germany) with using a 32-channel phased-array cardiac coil. All images were acquired with electrocardiogram (ECG)-gated breath-hold technique. All patients were scanned according to the standard imaging protocol for Ebstein’s anomaly [23]. The protocol consisted of stacks of balanced steady-state free-precession (bSSFP) cine images acquired in the three long-axis (two-, three-, and four-chamber) views, consecutive short-axis views covering the LV from base to apex (TR/TE 3.4 ms /1.3 ms, flip angle 50°, FOV 300 × 340 mm, matrix size 256 × 144, slice thickness 8 mm), and consecutive axial cine bSSFP images covering the whole heart extending from the pulmonary bifurcation to just below the diaphragm. LGE images were acquired using a T1-weighted inversion recovery turboflash sequence 10 to 20 min after bolus contrast injection (Gadopentetate dimeglumine, 0.15 mmol/kg, Bayer HealthCare Pharmaceuticals, Wayne, New Jersey, USA) in the same planes. T1 measurements were performed by using a Modified Look-Locker Inversion Recovery sequence (MOLLI) at the LV basal-, mid- and apical- levels, with the following parameters: non-selective inversion pulse, bSSFP single shot readout with 35° flip angle, minimum inversion time 110 ms, inversion time increment 80 ms, TR/TE of 2.9/1.12 ms, FOV of 360 × 272 mm, matrix size of 256 × 144, Voxel size of 2.1 × 1.4 × 8 mm^3^ and slice thickness of 8 mm. The inversion time for each patient was chosen at the time of scanning from the TI scout images, with three heartbeats for recovery between each experiment. Native T1 measurements were acquired before injection of gadolinium, with an imaging scheme of 5(3)3), and post contrast T1 measurements were repeated in the same short-axis slices 10–15 min after the administration of gadolinium with a scheme of 4(1)3(1)2. The hematocrit was tested within 24 h of CMR scanning for ECV calculation.

### CMR functional analysis

CMR images were analyzed using a commercially available software package (QMass® 7.6, Medis, Leiden, The Netherlands). Biventricular volume and function were obtained by tracing epicardial and endocardial contours manually on both end-diastolic and end-systolic phases. The LV volume, function, and mass were measured based on the consecutive short-axis slices. RV volume and function were derived from the axial views, as described in previous studies [1, 24]. All volumetric parameters were indexed to body surface area (BSA). The tracing method for the chambers was similar to the previous description by Fratz et al. [2, 25]. A line along the tricuspid leaflets was used to segment the aRV and the functional right ventricle (fRV). The fRV is defined as the part of the RV distal to the tricuspid leaflets. The border demarcating the aRV and morphological right atrium (RA) is defined as the curved line along the presumed tricuspid valve annulus, from the free wall attachment of the anterior tricuspid valve leaflet to the point of presumed septal leaflet attachment [25]. The trabeculae and papillary muscles were included in the ventricular blood pool.

### Quantification of myocardial fibrosis

LGE images were visually assessed on phase sensitive inversion recovery (PSIR) images at the short- and long-axis views by two experienced CMR imagers (YCC, and JYS, both with > 300 cases/year experience in CMR). The myocardial T1 calculation was based on the LV short axis MOLLI images with motion correction. The endocardial and epicardial contours were traced manually on the pre-contrast T1-mapping images and post-contrast T1-mapping images. The T1 curve was fitted automatically using the software (QMass® 7.6, Medis), and the mean myocardial T1 was obtained. At the same time, the blood T1 was obtained by drawing an ROI in the blood pool within the LV cavity in the pre-contrast and post-contrast T1-mapping images, respectively. The ECV was calculated as [26]:

$$ \mathrm{ECV}=\left(1-\mathrm{hematocrit}\right)\frac{\left(\frac{1}{T1\  myo\  post}-\frac{1}{T1\  myo\  pre}\right)}{\left(\frac{1}{T1\  blood\ post}-\frac{1}{T1\  blood\  pre}\right)} $$

### Severity of Ebstein’s anomaly

In our study, the severity index (SI) and total right/left-volume index (Total R/L-volume index) were utilized to quantify the severity of EA. SI, described by Fratz et al., was calculated using the area of the RA, aRV, fRV, left atrium (LA), and LV traced in end-diastole on the four-chamber view [1, 2, 25]. The total R/L-volume index was defined by Hösch et al. as the ratio of the total volume of the right heart and left heart, measured in the end-diastole on the axial views [27]. The equations for the two indices are shown as:

$$ \mathrm{SI}=\mathrm{area}\ \mathrm{of}\ \left(\frac{RA+ aRV}{fRV+ LA+ LV}\right) $$

Total R/L-Volume Index = volume of $\left(\frac{RA+ aRV+ fRV}{LA+ LV.}\right)$

### Statistical analysis

Statistical analysis was performed using SPSS 17.0 (SPSS Inc., International Business Machines, Armonk, New York, USA). Numerical data in normal distribution were expressed as the mean ± standard deviation (SD), or else were expressed as the median with the interquartile range. Continuous data were compared using a paired t-test. Categorical data were expressed as frequencies and compared using a χ^2^ test or the Fisher’s exact test as appropriate. Correlations between variables were analyzed by using Pearson’s or Spearman correlation analyses. Univariable factors related to the ECV at *p* <  0.1 were included in the multivariate stepwise regression analysis. Statistical significance was defined as *P* <  0.05.

## Results

### Demographic, clinical characteristics and basic CMR functional data

Ultimately, the Ebstein’s anomaly registry database comprised data from seventy-eight patients. Patients were excluded from the study because of: age < 10 years (*n* = 12), no contrast was used (*n* = 2), poor image quality caused by due to persistent atrial fibrillation or unable to breath hold (*n* = 10), and T1 mapping not performed (n = 10). The remaining 44 patients were included in the study. The mean age of the patients was 34.0 ± 16.2 years (range 10–60) years. Seven patients were between 10 and 18 years. Age, gender and BMI were well balanced between patients and the healthy control groups. Among the 44 patients, 29 (65.9%) had an atrial septal defect or patent foramen ovale. Tricuspid regurgitation was graded as severe in 32 (72.7%) and moderate in 12 (27.3%) patients (Table 1).

**Table 1:** Characteristics of patients and controls

	Ebstein’s anomaly(*N* = 44)	Healthy Subjects(*N* = 44)	*P* value
Age, yrs	34.0 ± 16.2	33.3 ± 16.1	0.923
10 ≤ age ≤ 18, yrs	7(15.9)	7 (15.9)	1.000
Male (n, %)	18(40.9)	18 (40.9)	1.000
BSA, m^2^	1.5 ± 0.2	1.6 ± 0.2	0.468
BMI, kg/m^2^	21.8 ± 4.8	21.7 ± 3.5	0.932
Heart rate, bpm	83.3 ± 12.7	73.6 ± 11.6	< 0.001
SO_2_ at rest_,_ %	96.6 ± 2.6	99.0 ± 1.0	< 0.001
Hematocrit, %	42.5 ± 4.9	44.1 ± 5.2	0.071
ASD/PFO (n, %)	29 (65.9)	0	–
W-P-W (n, %)	6 (13.6)	0	–
Tricuspid regurgitation severity (n, %)			–
Moderate	12 (27.3)	0	–
Severe	32 (72.7)	0	–
Carpentier’s classification			
Type A	18 (40.9)	–	–
Type B	17 (38.6)	–	–
Type C	8 (18.2)	–	–
Type D	1 (2.3)	–	–
NYHA functional classification (n, %)			
I	10 (22.7)	–	–
II	29 (65.9)	–	–
III	5 (11.4)	–	–

Abbreviations: *BSA* body surface area; *BMI* body mass index; *ASD* atrial septal defect; *PFO* patent foramen ovale; *W-P-W* Wolff-Parkinson-White; *NYHA* New York Heart Association

### Left and right ventricular volume, function and remodeling

LV end diastolic volume (EDV) index was reduced in Ebstein’s anomaly patients (68.3 ± 16.8 vs. 79.0 ± 17.1 ml, *P* = 0.007) (Table 2). LV ejection fraction (EF) and LV mass index in Ebstein’s anomaly was decreased (52.8 ± 7.6%, vs. 60.6 ± 3.7%, *P* <  0.001; and 39.2 ± 9.1 g/m2 vs. 45.2 ± 11.1 g/m2, *P* = 0.004, respectively). LVEF was reduced (< 50%) in 34.1% (*n* = 15) of the Ebstein’s anomaly patients. The LV mass to volume ratio was not significantly different from the control group. Both total and fRV EDV indices and RV end systolic volume (ESV) index were significantly increased compared with those of the controls. The fRV EF in Ebstein’s anomaly was significantly reduced (*P* <  0.001).

**Table 2:** Cardiovascular magnetic resonance parameters

	Ebstein’s anomaly(*N* = 44)	Healthy subjects(*N* = 44)	*P* value
LV EDV index, ml/m^2^	68.3 ± 16.8	79.0 ± 17.1	0.007
LV ESV index, ml/m^2^	32.5 [12.1]	31.3 [7.9]	0.577
LV EF, %	52.8 ± 7.6	60.6 ± 3.7	< 0.001
LV mass index, g/m^2^	39.2 ± 9.1	45.2 ± 11.1	0.004
LV mass/volume	0.58 ± 0.15	0.60 ± 0.10	0.892
fRV EDV index, ml/m^2^	138.9 [66.6]	73.6 [18.6]	< 0.001
fRV ESV index, ml/m^2^	77.3 [41.9]	34.4 [10.4]	< 0.001
fRV EF, %	43.7 ± 11.5	53.6 ± 6.3	< 0.001
Total R/L-Volume-Index	4.3 ± 2.5	–	–
SI	1.0 ± 0.4	–	–
LV Native T1, ms			
Global	1224.8 ± 86.6	1198.8 ± 45.4	0.084
Septal	1244.4 ± 97.1	1218.0 ± 44.3	0.124
Free wall	1209.5 ± 103.8	1199.9 ± 71.2	0.630
LV ECV, %			
Global	30.0 ± 3.8	25.3 ± 2.3	< 0.001
Septal	31.2 ± 5.0	25.4 ± 2.3	< 0.001
Free wall	28.9 ± 4.5	25.4 ± 2.7	< 0.001
LGE positive, n (%)	10 (22.7)	–	–

Abbreviations: *LV* left ventricle; *EDV* end diastolic volume; *ESV* end systolic volume; *EF* ejection fraction; *fRV* functional right ventricle; Total R/L-Volume-Index, total right/left volume index; *SI* severity index; *ECV* extracellular volume; *LGE* late gadolinium enhancement

### Prevalence, characteristics, and clinical relevance of LGE

**Fig. 1:**
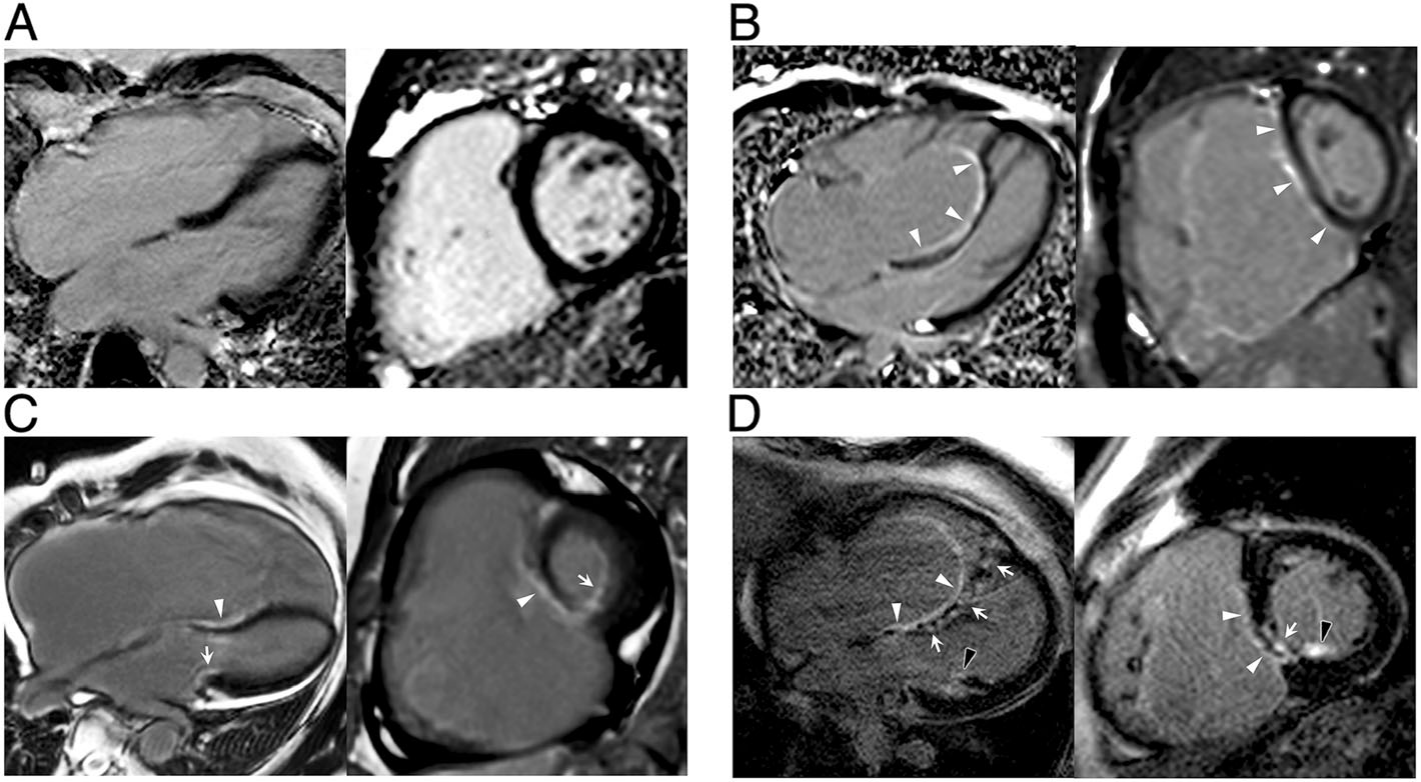
Representative images of late gadolinium enhancement (LGE) in patients with Ebstein’s anomaly. **a**. Patient without LGE. **b**. Endocardial LGE along atrialized right ventricular (aRV) wall (white arrow heads). **c**. LGE in both left ventricular (LV) (arrows) and aRV (white arrow heads) endothelium. **d**. Endocardial LGE in aRV (white arrow heads), with sporadic and patchy myocardial LGE in interventricular septum (arrows). LGE was also noted in the posterior papillary muscle (black arrow heads). LGE, late gadolinium enhancement; aRV, atrialized right ventricle; LV, left ventricle

LGE was seen in 10 out of 44 (22.7%) Ebstein’s anomaly patients. The typical LGE pattern was linear or patchy localized within the RV subendocardium of the basal septum (Fig. 1). Only one Ebstein’s anomaly patient showed focal transmural LGE in the basal septum and posteromedial papillary muscle (shown in Fig. 1). No remarkable LGE was seen in the LV free wall. In Ebstein’s anomaly patients with LGE, the indexed fRV EDV and ESV were significantly larger than those patients without LGE (both *P* value < 0.01). The fRV EF was lower in patients with LGE than without. The aRV volume was also significantly larger in the LGE-positive group than the LGE-negative group. Ebstein’s anomaly severity evaluated by the total R/L-volume index was more severe in patients with LGE than without LGE, while the SI was not significantly different between these two groups. Patients with LGE also had a higher NYHA functional class and lower oxygen saturation at rest than patients without LGE (*P* = 0.011 and 0.042, respectively). The LV volume and the LV EF were lower in patients with LGE; however, the difference was not significant. All data are shown in Table 3.

**Table 3:** Comparison between Ebstein anomaly patient groups with and without LGE

	LGE positive (*N* = 10)	LGE negative (*N* = 34)	*P*
Age, yrs	40.4 ± 16.1	31.6 ± 15.8	0.131
Male (n, %)	6 (60)	12 (35.3)	0.273
Heart rate, bpm	78.1 ± 7.8	84.7 ± 13.4	0.147
BMI, kg/m^2^	22.8 ± 4.8	21.4 ± 4.8	0.428
NYHA classification	2.3 ± 0.5	1.8 ± 0.5	0.011
SO_2_ at rest, %	95.1 ± 2.6	97.1 ± 2.5	0.042
Severe TR (n, %)	8 (80.0)	24 (70.6)	0.557
ASD/PFO (n, %)	7 (70.0)	22 (64.7)	1.000
W-P-W (n, %)	1 (10.0)	5 (14.7)	0.950
QRS width, ms	114.2 ± 19.0	113.5 ± 15.2	0.910
LV EDV index, ml/m^2^	62.5 [12.6]	71.1 [21.2]	0.233
LV ESV index, ml/m^2^	30.6 [5.6]	33.7 [15.3]	0.537
LV EF, %	49.2 ± 10.5	53.7 ± 6.7	0.106
fRV EDV index, ml/m^2^	194.5 [87.7]	127.4 [57.4]	0.007
fRV ESV index, ml/m^2^	123.6 [64.4]	67.4 [28.7]	< 0.001
fRV EF, %	40.1 ± 12.7	45.0 ± 11.1	0.251
aRV EDV index, ml/m^2^	107.6 ± 76.16	62.4 ± 35.9	0.011
Total R/L-Volume-Index	6.9 ± 3.4	3.5 ± 1.7	0.011
SI	1.1 ± 0.4	0.9 ± 0.4	0.195

Abbreviations: *LGE* late gadolinium enhancement; *BMI* body mass index; *NYHA* New York Heart Association; *TR* tricuspid regurgitation; *ASD* atrial septal defect; *PFO* patent foramen ovale; *W-P-W* Wolff-Parkinson-White; *LV* left ventricle; *EDV* end diastolic volume; *ESV* end systolic volume; *EF* ejection fraction; *fRV* functional right ventricle; *aRV* atrialized right ventricle; Total R/L-Volume-Index, total right/left volume index; *SI* severity index

### Average native T1, myocardial ECV, and regional changes

The native T1 was higher but not statistically significant in patients with Ebstein’s anomaly. The average myocardial ECV was significantly increased in patients with Ebstein’s anomaly (*P* < 0.001). The septal ECV was higher than that of the free ventricular wall in Ebstein’s anomaly patients and free wall ECV was significantly higher than that of the controls (P < 0.001). Twenty (45.5%) patients had an ECV higher than 30%, which is above the upper limits of normal. Except for one a-60-year old, all subjects in the control group had an ECV less than 30%. In patients with a higher ECV, the Ebstein’s anomaly severity index, NYHA class and tricuspid regurgitation were significantly worse than in patients with a normal ECV. The LVEF was much lower in Ebstein’s anomaly patients with a higher ECV than those with a normal ECV (Table 4). The hematocrit was observed to be lower in patients with higher ECV, while no linear correlation was present between the hematocrit and the ECV of the LV myocardium.

**Table 4:** Comparison between Ebstein anomaly patient groups with different ECV

	ECV ≥ 30%(*N* = 20)	ECV < 30%(*N* = 24)	*P*
Age, yrs	34.9 ± 15.7	32.6 ± 16.7	0.648
Male (n, %)	6 (30.0)	12 (50.0)	0.227
Heart rate, bpm	83.6 ± 11.0	83.0 ± 14.0	0.852
BMI, kg/m^2^	21.2 ± 4.3	22.2 ± 5.2	0.488
NYHA classification	2.1 ± 0.4	1.7 ± 0.6	0.020
SO_2_ at rest, %	96.2 ± 2.6	97.0 ± 2.8	0.411
Severe tricuspid regurgitation (n, %)	18 (90.0)	14 (58.3)	0.019
ASD/PFO (n, %)	15 (75.0)	14 (58.3)	0.342
W-P-W (n, %)	3 (15.0)	3 (12.5)	0.329
Hematocrit, %	40.0 ± 4.3	44.7 ± 4.5	0.001
QRS width, ms	113.2 ± 17.3	114.1 ± 14.9	0.862
LV EDV index, ml/m^2^	67.7 [26.0]	70.3 [13.2]	0.671
LV ESV index, ml/m^2^	35.6 [18.7]	31.0 [7.1]	0.259
LV EF, %	48.0 ± 8.3	56.7 ± 4.7	< 0.001
fRV EDV index, ml/m^2^	168.1 [77.7]	121.4 [56.8]	0.026
fRV ESV index, ml/m^2^	100.0 [53.7]	63.6 [29.3]	0.011
fRV EF, %	38.4 ± 12.3	46.2 ± 11.0	0.117
aRV EDV index, ml/m^2^	89.0 ± 58.4	59.1 ± 39.7	0.051
Total R/L-Volume-Index	5.8 ± 2.8	3.1 ± 1.4	< 0.001
SI	1.13 ± 0.4	0.86 ± 0.4	0.036

Abbreviations: *ECV* extracellular volume; *BMI* body mass index; *NYHA* New York Heart Association; *ASD* atrial septal defect; *PFO* patent foramen ovale; *W-P-W* Wolff-Parkinson-White; *LV* left ventricle; *EDV* end diastolic volume; *ESV* end systolic volume; *EF* ejection fraction; *fRV* functional right ventricle; *aRV* atrialized right ventricle; Total R/L-Volume-Index, total right/left volume index; *SI* severity index

**Fig. 2:**
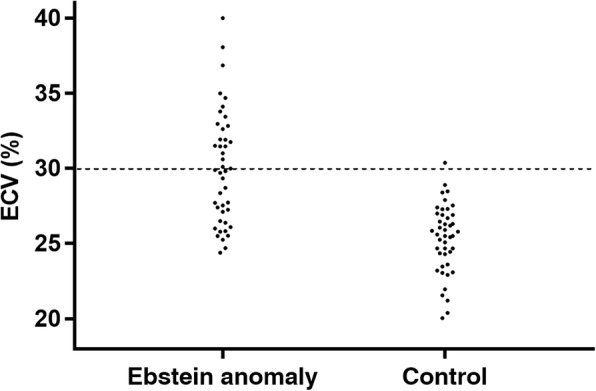
Distribution of global extracellular volume (ECV) in patients with Ebstein’s anomaly and healthy control subjects. ECV, extracellular volume

**Fig. 3:**
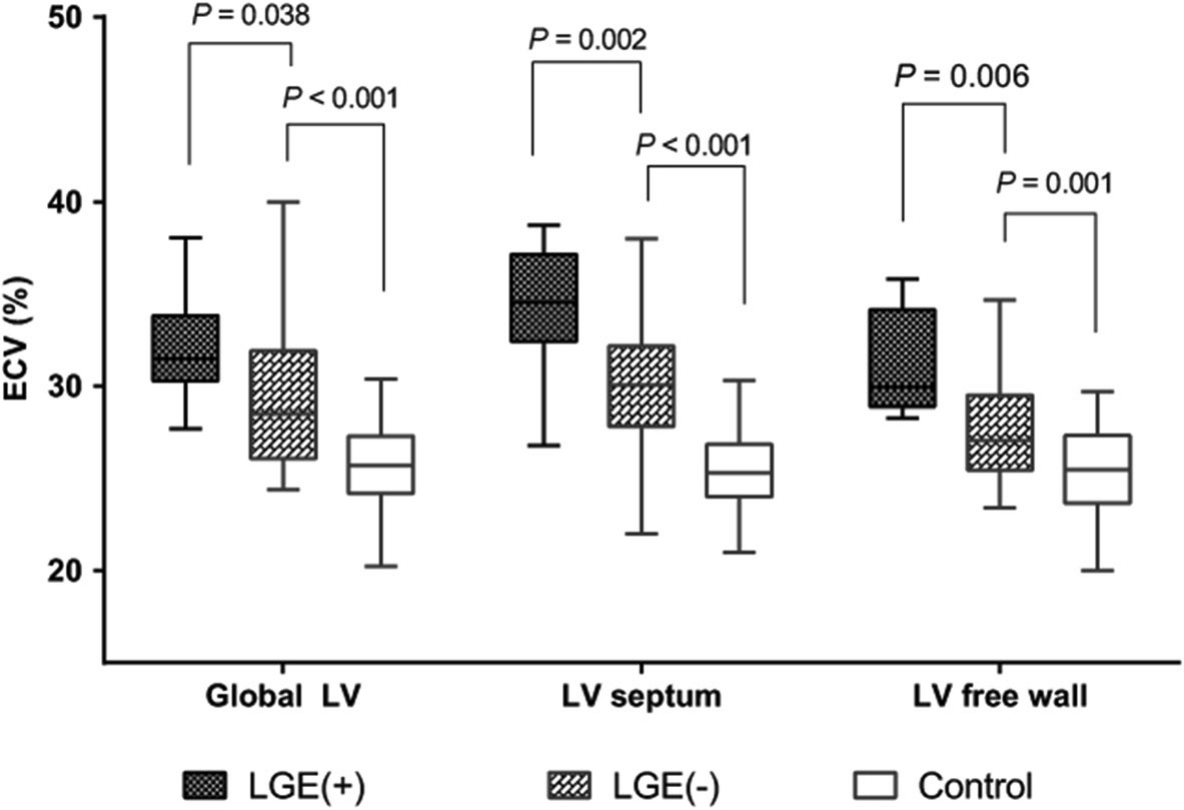
Box plots display the discrepancy of ECV among LGE (+), LGE (−) and healthy control group in global, septal wall and LV free wall. ECV, extracellular volume; LGE, late gadolinium enhancement; LV, left ventricle

To explore the relationship between LGE and the ECV, we compared ECV between Ebstein’s anomaly patients with and without LGE. Figure 2 shows that the global ECV was higher in Ebstein’s anomaly patients with LGE than patients without LGE (*P* = 0.038). It is important to recognize that the ECV in patients without LGE was still significantly higher than that of the controls. The same trend was found (Fig. 2) when the ECV was analyzed separately in the septum versus the free wall. Individual global ECV values in the EA and control groups are shown in Fig. 3.

### Correlation between the ECV and clinical characteristics, ventricular function and disease severity

Among all variables, the ECV of the LV myocardium was significantly correlated with NYHA class, tricuspid regurgitation severity, the LV ESV index, the LV EF, the fRV EDV index, the fRV ESV index, and the fRV EF (Table 5). Moreover, we found that the ECV of the LV myocardium had a moderate correlation with the Ebstein’s anomaly severity (severity index: *r* = 0.392, *P* = 0.009; Total R/L-volume index: *r* = 0.612, *P* < 0.001). Multivariate correlation analysis showed that ECV was independently correlated with LVEF.

**Table 5:** Univariate and multivariate linear regression of factors associated with ECV

	Univariate		Multivariate		
	r	*P*		B	*P*
Age	−0.043	0.782			
Gender	−0.058	0.707			
NYHA classification	−0.407	0.006		0.082	0.518
SO_2_	−0.195	0.093			
Hematocrit, %	−0.282	0.163			
Tricuspid regurgitation severity	−0.326	0.031		0.022	0.858
ASD/PFO	0.145	0.346			
W-P-W, %	−0.021	0.893			
LV EDV index	0.132	0.392			
LV ESV index	0.415	0.005			
LVEF	−0.694	< 0.001		−0.330	< 0.001
fRV EDV index	0.407	0.006		0.123	0.326
fRV ESV index	0.470	0.001			
fRV EF	−0.307	0.043		−0.054	0.658
aRV EDV index	0.274	0.072			
Total R/L-volume index	0.612	< 0.001		0.210	0.215
SI	0.392	0.009		0.061	0.470

Abbreviations: *ECV* extracellular volume; *NYHA* New York Heart Association; *SI* severity index; *ASD* atrial septal defect; *PFO* patent foramen ovale; *W-P-W* Wolff-Parkinson-White; *LV* left ventricle; *EDV* end diastolic volume; *ESV* end systolic volume; *EF* ejection fraction; *fRV* functional right ventricle; *aRV* atrialized right ventricle; Total R/L-Volume-Index, total right/left volume index; *SI* severity index

## Discussion

To the best of our knowledge, this is the first study to investigate diffuse myocardial fibrosis in Ebstein’s anomaly using CMR. The main findings are: (1) both focal myocardial fibrosis and diffuse myocardial fibrosis were detected by CMR in Ebstein’s anomaly patients. (2) Diffuse myocardial fibrosis was more common than focal myocardial fibrosis in Ebstein’s anomaly, and both were associated with the severity of Ebstein’s anomaly. (3) Diffuse myocardial fibrosis demonstrated by T1 mapping was closely associated with LV dysfunction.

Ebstein’s anomaly has a wide spectrum of clinical manifestations, from early death in neonates to mild symptoms surviving into adulthood [28]. Heart failure is one of the common symptoms in adult Ebstein’s anomaly, which is a result of biventricular dysfunction [7, 29–31]. LV dysfunction was observed in previous studies and is considered a high-risk factor for mortality after surgery and long-term survival [10, 32]. In Ebstein’s anomaly, LV dysfunction was considered as a consequence of compression caused by a dilated RV and abnormal septal motion [9]. A recent study further corroborated LV dysfunction in Ebstein’s anomaly with LV regional irregularity and the effect of RV longitudinal contraction. However, these mechanisms do not adequately explain why in some patients, postoperative LV dysfunction persisted and was associated with suboptimal survival [10].

The finding of LV myocardial fibrosis in Ebstein’s anomaly provides another perspective on this question. In early studies, myocardial fibrosis was found in ex-vivo hearts of patients with Ebstein’s anomaly, in which histopathological examination demonstrated myocardial fibrosis existed in the fetus and neonate. Interstitial fibrosis was more common in both the RV LV and sporadic replacement myocardial fibrosis also existed [33–35]. Whether or not myocardial fibrosis is an intrinsic property or an acquired characteristic secondary to abnormal hemodynamics in Ebstein’s anomaly is still controversial [33]. Only one study showed that the heart in adult Ebstein’s anomaly patients had interstitial myocardial fibrosis in both ventricles, while very mild myocardial fibrosis was shown in neonates. Due to the limited specimen from autopsies, it is very difficult to confirm detailed mechanisms and characteristics of myocardial fibrosis in Ebstein’s anomaly.

In the present study, both LGE and elevated ECV were observed in Ebstein’s anomaly patients. The typical LGE pattern found in this study was mostly localized in the endocardium of the aRV septum. This finding was in accordance with a previous case report [36]. In one case, mid myocardial wall LGE (non-ischemic pattern) was observed in the basal to mid septum. In contrast to LGE, which was only found in a minority of Ebstein’s anomaly patients, interstitial fibrosis, as evidenced by a mean LV ECV elevation greater than the upper two SD limits of the control ECV, was demonstrated in more than 45% of patients. This finding was in line with previous autopsy results [33].

Both LGE and elevated ECV were related to the severity of Ebstein’s anomaly and ventricular dysfunction in this group of Ebstein’s anomaly patients. This finding emphasizes the importance of focal and diffuse myocardial fibrosis in ventricular remodeling in Ebstein's anomaly. LV remodeling was related to Ebstein’s anomaly severity, which demonstrated a close RV-LV interaction [37, 38]. Furthermore, the relationship between the severity of Ebstein’s anomaly and LV ECV also suggested the impact of an abnormal RV on LV myocardial remodeling [10]. The inverse relationship between an elevated LV ECV and LV function found in Ebstein’s anomaly patients was observed in dilated cardiomyopathy [39]. LV dysfunction in Ebstein’s anomaly has been considered to result from chronic hemodynamic under-filling of the LV and is possibly related to myocardial cell degeneration. The present study provided evidence for diffuse myocardial fibrosis, which could be an important contributor to biventricular dysfunction in Ebstein’s anomaly.

LV dysfunction is a high-risk factor in Ebstein’s anomaly and is associated with higher mortality after surgery [40]. Preservation and reversal of LV dysfunction in Ebstein’s anomaly could be a potential treatment target. In patients with mild or asymptomatic Ebstein’s anomaly, indications for surgery include deterioration of ventricular function, persistent enlargement of the heart, exercise intolerance, and arrhythmia [41]. The value of CMR detection of myocardial fibrosis in Ebstein’s anomaly in decision-making for surgical treatment requires further study. In a study on aortic valve stenosis, an elevated ECV persisted for at least six months after aortic valve replacement surgery [42]. Earlier intervention might be necessary to reverse myocardial remodeling.

In our study, ECV was also associated with clinical and imaging-based ventricular function. Thus, ECV is a potential imaging marker for risk stratification in Ebstein’s anomaly patients. Future CMR evaluation for Ebstein’s anomaly should not only include volume and function analysis, but also consider myocardial tissue characterization using LGE and ECV.

### Limitations

There are several limitations to our study. First, the present study consisted of both adolescent and adult patients, and does not represent the entire Ebstein’s anomaly spectrum. Second, the ECV of the RV myocardium was not quantified because the RV free wall was too thin in Ebstein’s anomaly for accurate assessment. Third, the cross-sectional study design limited the ability to determine the impact of myocardial fibrosis on the long-term prognosis of Ebstein’s anomaly. Our study indicated that further longitudinal observation is needed to clarify the impact of myocardial fibrosis and remodeling in Ebstein’s anomaly patients.

## Conclusions

Focal and diffuse myocardial fibrosis assessed by LGE and ECV were observed in Ebstein’s anomaly patients. The elevated fibrosis was associated with worse LV function, greater disease severity, and reduced clinical status in Ebstein’s anomaly patients. LGE or ECV may serve as imaging markers for the severity of Ebstein’s anomaly. Further studies are needed to assess the prognostic significance of these findings.

## Abbreviations

aRV: Atrialized right ventricle
BSA: Body surface area
bSSFP: Balanced steady state free precession
CMR: Cardiovascular magnetic resonance
ECG: Electrocardiogram
ECV: Extracellular volume
EDV: End-diastolic volume
EF: Ejection fraction
ESV: End-systolic volume
fRV: Functional right ventricle
LA: Left atrium/left atrial
LGE: Late gadolinium enhancement
LV: Left ventricle/left ventricular
MOLLI: Modified Look-Locker inversion recovery
PSIR: Phase sensitive inversion recover
RA: Right atrium/right atrial
ROI: Region of interest
RV: Right ventricle/right ventricular
SI: Severity index
Total R/L-volume index: Total right/left volume index
